# Similarities in the Neural Control of the Shoulder and Elbow Joints Belie Their Structural Differences

**DOI:** 10.1371/journal.pone.0045837

**Published:** 2012-10-17

**Authors:** Andrew R. Karduna, Robert L. Sainburg

**Affiliations:** 1 Department of Human Physiology, University of Oregon, Eugene, Oregon, United States of America; 2 Department of Kinesiology and Neurology, Pennsylvania State University, University Park, Pennsylvania, United States of America; The University of Western Ontario, Canada

## Abstract

Movement of the hand in three dimensional space is primarily controlled by the orientation of the shoulder and elbow complexes. Due to discrepancies in proprioceptive acuity, overlap in motor cortex representation and grossly different anatomies between these joints, we hypothesized that there would be differences in the accuracy of aimed movements between the two joints. Fifteen healthy young adults were tested under four conditions – shoulder motion with the elbow constrained and unconstrained, and elbow motion with the shoulder constrained and unconstrained. End point target locations for each joint were set to coincide with joint excursions of 10, 20 or 30 degrees of either the shoulder or elbow joint. Targets were presented in a virtual reality environment. For the constrained condition, there were no significant differences in angular errors between the two joints, suggesting that the central nervous system represents linked segment models of the limb in planning and controlling movements. For the unconstrained condition, although angle errors were higher, hand position errors remained the same as those of the constrained trials. These results support the idea that the CNS utilizes abundant degrees of freedom to compensate for the potentially different contributions to end-point errors introduced by each joint.

## Introduction

The ability of the central nervous system (CNS) to position the hand at specific locations in the workspace is a critical component of most activities of daily living and is required for reaching to procure and manipulate objects. Previous studies have shown that the accuracy and variability of end point positioning of the hand is dependent on numerous factors, including arm dominance [1], movement velocity [2], joint excursion [3], [4], initial positioning [5], [6], age [7], and proprioceptive information [8], [9]. While the whole body can be involved in this task, accurate and repeatable positioning of the hand is predominantly controlled by motion of the shoulder and elbow joints. Consequently, understanding the relative accuracy of these two joints is important for a better understanding of how we position our hands in space. There are three key factors that determine this accuracy: sensory processing, motor control and joint anatomy.

From a sensory point of view, the question of which joint is more accurate was first introduced over a century ago by Goldscheider [10], who observed that proprioceptive acuity is better at the shoulder when using a threshold for detecting passive motion model. More recent studies have produced mixed results. Using a similar model as that of Goldscheider, Hall and Smith [11] found no difference between the two joints, while Sturnieks and Fitzpatrick [12] reported a higher detection capability at the elbow, which the authors attributed to different levels of muscle spindle preconditioning in their protocol. However, using active joint repositioning tasks, both Clark et al. [13] and Tripp et al. [14] observed higher accuracy at the shoulder. Overall, these comparisons of proprioception between the shoulder and elbow provide inconclusive data regarding which joint is more accurate.

In their pioneering work on developing the motor homunculus, Penfield and Rassmussen [15] showed that representations of shoulder and elbow joint movements in motor cortex are similar in size. This was confirmed quantitatively in a recent fMRI study by Kocak et al. [16], in which similar activation volumes in the precentral gyri were observed when subjects were asked to actively flex either their elbow or shoulder. However, it should be stressed that while the mechanical features of the upper extremity can be represented as a linked-segment inertial system, many sensors and actuators span multiple segments. As a result, the joints are not necessarily represented independently by the CNS and there is clearly overlap in motor cortex between these two joints [17], [18], [19]. Additionally, about a third of the neurons in the shoulder/elbow region of M1 show activity that is related to torque generation at both joints [20].

With respect to joint anatomy, there are many factors that could result in differing joint accuracies. The elbow is a fairly simple, two degree of freedom hinge joint for which the inherent joint stability is provided by the bony morphology of the joint. In contrast, the shoulder complex is comprised of three diarthrodial joints, each with three degrees of rotational freedom and the stability of the glenohumeral joint is mainly provided by muscular contractions. Additionally, the shoulder has more muscles and an overall larger muscle mass [21]. There is also a dramatic difference in the lever arm between the joint centers and the hand, as well as the inertial resistance at each joint. Interestingly, the number of muscle spindles around each joint appears to be similar [22].

Given plausible discrepancies in proprioceptive acuity, overlap in motor cortex representation and grossly different anatomies, it seems reasonable to hypothesize that differences in positional accuracy should occur between movements of the shoulder and elbow joints. We have designed an experiment to answer two fundamental questions related to shoulder and elbow motion. The first question asks whether there are differences in the accuracy of aimed movements between the shoulder and elbow, both in terms of joint angles and end point positions. This question will be answered by studying the motion of each joint in isolation. The second question asks whether there are differences in the accuracy of aimed movements between single and multi-joint tasks. This question will be answered by comparing motions where only one joint is free to move with motions in which both joints are unconstrained.

## Methods

### Subjects

Fifteen neurologically intact subjects (5 males, 10 females) with a mean body mass of 70 (+/−12) kg and a mean height of 170 (+/−10) cm agreed to participate in this study. Handedness was determined using a 35-item version of the Edinburgh inventory [23], and only those classified as right-handers were used for the experiment. In general, subjects were healthy college students. The Institutional Review Board of the Pennsylvania State University specifically approved this study. Prior to testing, written informed consent was obtained from all participants.

### Experimental setup

**Figure 1:**
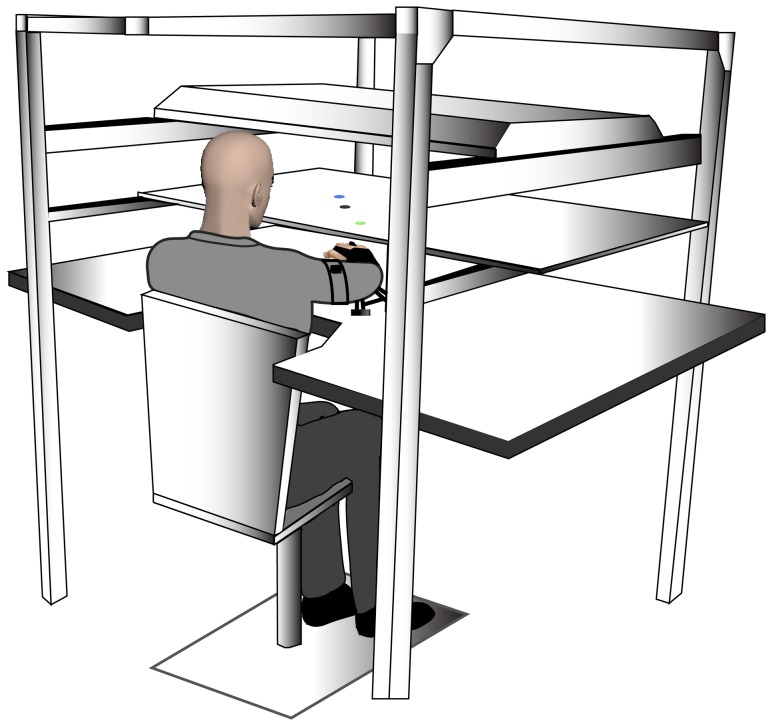
Schematic representation of experimental set-up. The subject is seated with electromagnetic sensors placed on their arm and forearm. Vision of the upper extremity is blocked and targets are presented through a 2D virtual reality environment.

Subjects were tested using a virtual reality type environment (Figure 1). They were seated with their right upper extremity over a table surface that was positioned just below shoulder height. The arm and forearm were supported by an air-sled system designed to reduce the effects of friction. The trunk was stabilized and all joints distal to the elbow were immobilized with a brace. A mirror positioned above the table reflected images from a 52 inch flat screen TV (Sony), which received inputs from a computer. The system was able to display the position of the index finger interphalangeal joint (3.5 cm diam), a start position (3.5 cm diam) and target position (3.5 cm diam). Calibration of the display assured that projections on the mirror were veridical. Position and orientation of the forearm and arm segments were recorded using a Flock of Birds electromagnetic system (Ascension Technology) at a sampling frequency of 130 Hz. One sensor was attached to the arm segment by means of a plastic arm cuff and another sensor was attached to the air sled on which the forearm rested. The sensors were positioned near the midpoint of each segment. The positions of the interphalangeal joint, lateral epicondyle, wrist center and posterior acromion were digitized using a stylus that was rigidly attached to another sensor. As data were received from the sensors, the 3-D position of the above-mentioned landmarks were computed using custom software, with the *x–y* plane parallel to the tabletop. The *x–y* coordinates of the interphalangeal joint were used to define the projected cursor position. Custom computer algorithms for experimental control and data analysis were written in REAL BASIC (REAL Software) and Igor Pro (Wavemetrics), respectively.

The induced constraints of the experimental setup resulted in two degrees of rotational freedom, one for the shoulder and one for the elbow, both in the transverse plane. Motion of the shoulder and elbow resulted in two degrees of translational freedom of the end effector (hand). By matching the numbers of degrees of freedom, there was a one to one mapping between the joint space and end effector space. Given the length of the arm and forearm, a set of equations were developed using the Denavit-Hartenberg convention so that joint angles could be used to calculate end effector positions and visa versa.

### Experimental task

There were four experimental sessions: 1) shoulder targets with the elbow constrained; 2) elbow targets with the shoulder constrained; 3) shoulder targets with the elbow unconstrained; and 4) elbow targets with the shoulder unconstrained. For the shoulder constrained session, the arm was secured to the table with a brace and suction cup. For the elbow constrained session, the elbow was immobilized with an IROM Elbow Brace (DonJoy Orthpedics). In order to allow for the upper extremity to fit in the air-jet system, this brace was modified to remove most of the plastic supports. For the unconstrained sessions, no braces were used (except for the wrist brace). The starting position for all trials was at 50 degrees of shoulder horizontal abduction and 50 degrees of elbow flexion. Shoulder targets were generated at 60, 70 and 80 degrees of shoulder horizontal abduction (with a constant elbow flexion angle). Similarly, elbow targets were generated at 60, 70 and 80 degrees of elbow flexion (with a constant shoulder abduction angle). In other words, targets were designed to isolate 10, 20 and 30 degrees of joint excursion of either the shoulder or elbow.

Because of anthropometric differences, subject-specific target x, y positions were generated. Additionally, to account for potential changes in trunk position, new targets were generated at the beginning of each session. Each target in each session was presented a total of 10 times, yielding 30 trials per session and 120 trials per subject. Targets were presented in a pseudorandom order so that no single target was presented consecutively. Session order was randomized as to whether the shoulder or elbow targets were first and whether the constrained or the unconstrained trials were first.

Prior to each trial, a start position was displayed. Subjects positioned their upper extremity so that their interphalangeal joint was located in this target. After 300 ms, a target position was presented and beep sounded, indicating that subjects should move their hand towards the target. Subjects were asked to make a “rapid, but direct movement” to the projected target. There was no feedback with regard to the location of their hand at any time during the trial.

### Kinematic Data Analysis

The 2-D position of the wrist, elbow and shoulder were calculated from sensor position and orientation data. Shoulder and elbow joint angles were calculated from these data. All kinematic data were low-pass filtered at 8 Hz (3rd order, dual-pass Butterworth). End position of the hand (index finger interphalangeal joint) was defined as the first minimum (<8% maximum tangential hand velocity) after the peak in tangential hand velocity.

Angular errors were calculated as described by Schmidt and Lee. [24] The angular constant error for each trial was calculated as the difference between the end angle (θ_i_) and the target angle (θ_t_). The data were then averaged over the ten trials (n) for that target.
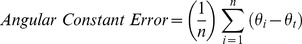
(1)The angular variable error for each trial was calculated as the difference between the end angle (θ_i_) and the mean angle for the 10 trials for that target (θ_m_). The data were then squared, averaged over the ten trials (n) for that target and the square root of that average was calculated.
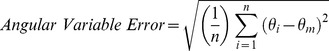
(2)Linear errors were calculated as described by Hancock et al. [25] Linear constant error (mean radial error) for each trial was calculated as the linear distance from the end position x_i_, y_i_ coordinates to the target x_t_, y_t_ coordinates. The data were then averaged over the ten trials (n) for that target.
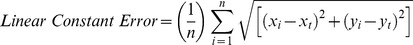
(3)Linear variable error (bivariate variable error) for each trial was calculated as the distance from the end position x_i_, y_i_ coordinates to the mean end point x_m_, y_m_ coordinates for the ten trials (n) for that target. The data were then averaged over the ten trials for that target.
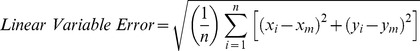
(4)


### Statistical Analysis

Statistical analyses were performed using SPSS, version 18 (IBM Corporation, Somers, NY). In order to answer our first research question regarding the difference between shoulder and elbow joint motions, a repeated measures ANOVA was run on the constrained data with two within subject factors: angles (10, 20, 30) and joint (shoulder, elbow). This analysis was run on both the linear and angular data. For the angular data, we only analyzed the unconstrained joint (eg, elbow when the shoulder was locked and shoulder when the elbow was locked). In order to answer our second research question regarding the effects of joint constraint, a repeated measures ANOVA was run with two within subject factors: angles (10, 20, 30) and motion (constrained, unconstrained). This analysis was run for both the elbow and shoulder targets. For the linear data, this was straightforward, as there was a single error term for each trial. However, since we were now interested in both joints for all trials, we ran a separate angular analysis for the motions of the shoulder and elbow. For all analyses, when there were no significant interactions, the main effects are presented directly. Where there were significant interactions, follow up t-tests were run with an appropriate Bonferroni correction to account for multiple comparisons. The alpha level was set at 0.05 for all analyses.

## Results

### Shoulder vs. Elbow – Constant Errors

**Figure 2:**
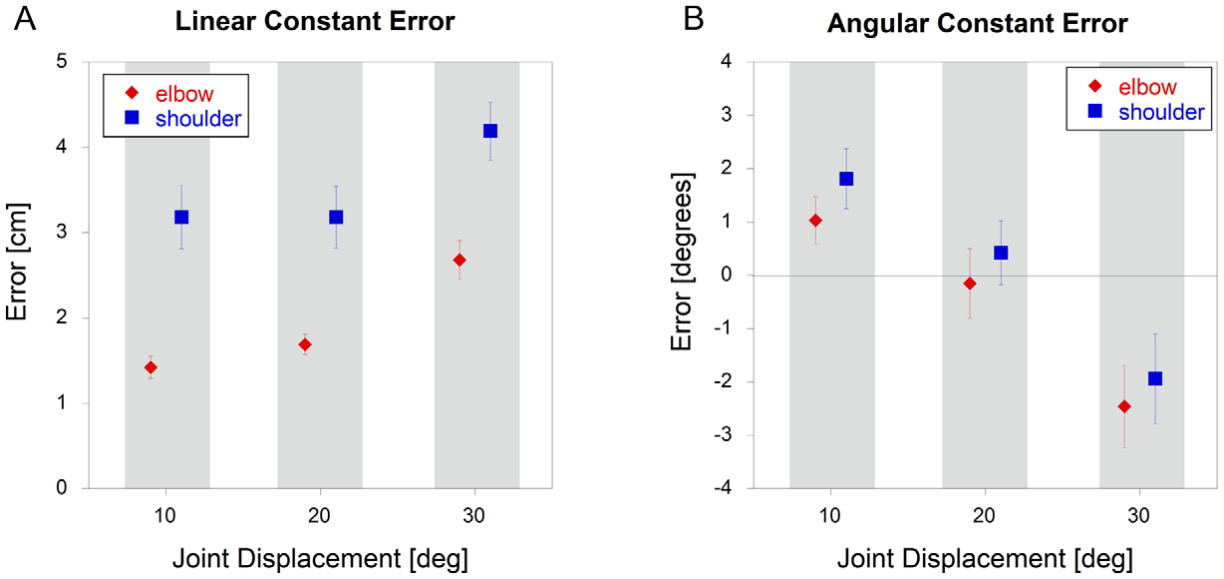
Constant errors for single joint motions of the shoulder and elbow (mean +/− sem). Errors were calculated using both A) linear and B) angular data.

The linear constant error was significantly higher (p<0.001) for the shoulder targets than for the elbow targets, with a gradual increase in errors (p<0.001) with increasing joint displacement (Figure 2a). However, when the data were re-analyzed in terms of angular errors, there was no significant difference (p = 0.36) between the shoulder and elbow targets (Figure 2b). There was also a significant effect of joint displacement (p<0.001), with subjects overshooting the target at low displacements and undershooting the targets at high displacements.

### Shoulder vs. Elbow – Variable Errors

**Figure 3:**
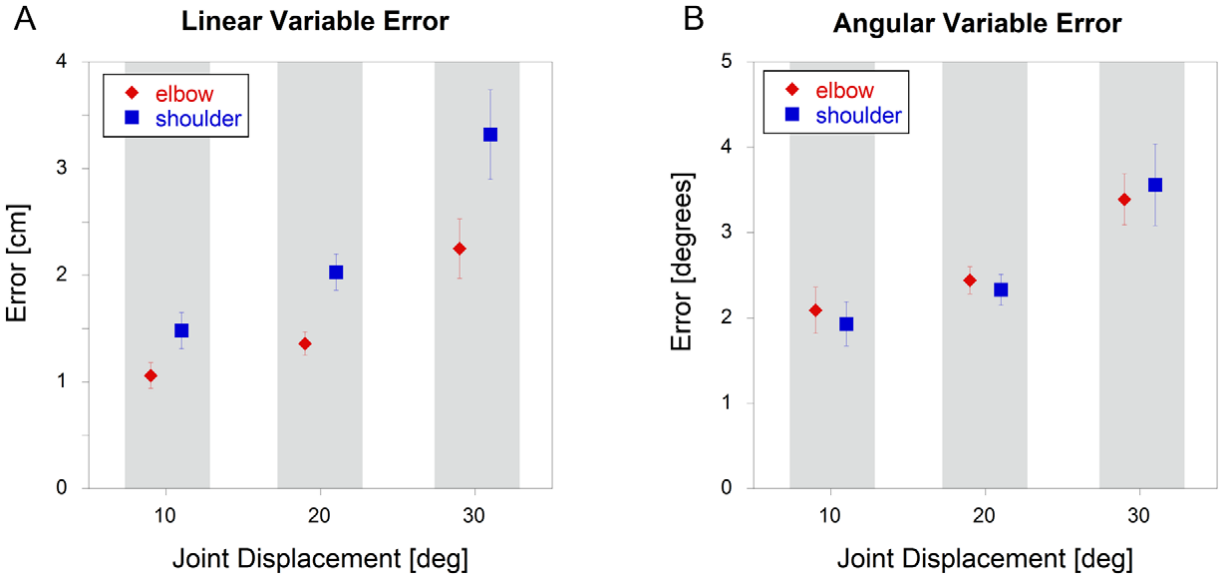
Variable errors for single joint motions of the shoulder and elbow (mean +/− sem). Errors were calculated using both A) linear and B) angular data.

As with constant errors, linear variable errors were significantly higher (p<0.001) for the shoulder targets when compared to the elbow targets (Figure 3a). Similarly, these difference were abolished (p = 0.88) when the data were analyzed in terms of joint angles (Figure 3b). For both angular and linear analyses, there was a significant increase in errors (p<0.001) as joint displacement increased.

### Constrained vs. Unconstrained - Constant Errors

**Figure 4:**
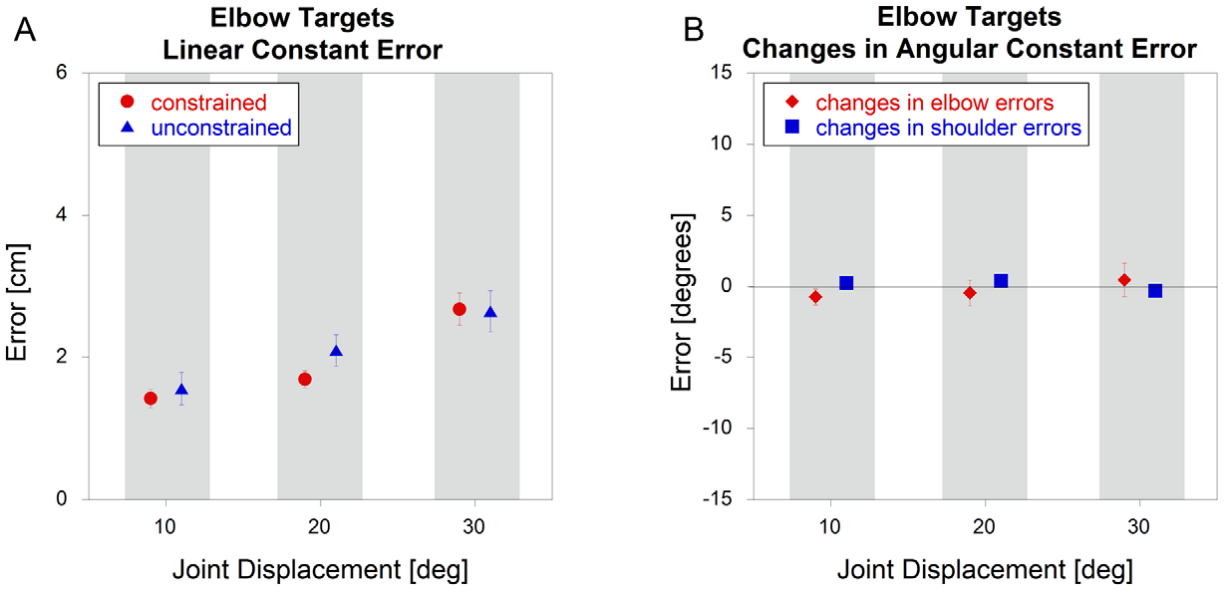
Elbow target constant errors comparing constrained and unconstrained conditions (mean +/− sem). The data represent A) linear constant errors and B) *changes* in angular constant errors. NOTE - some of the error bars are too small to be seen.

For elbow targets, there was no significant effect (p = 0.44) of constraint on linear constant errors. When the data were analyzed in terms of joint angles, there were still no significant effects of constraint on the errors for both the elbow (focal) joint (p = 0.77) and shoulder (non-focal) joint (p = 0.76). There was a significant interaction with joint angle for shoulder angles, but follow-up t-tests revealed no effect (p>0.36) of constraint for movements to any target. So for the elbow targets, constant errors were no different for constrained or unconstrained conditions, regardless of whether the data were analyzed in terms of end point position (Figure 4a) or joint angles (Figure 4b).

**Figure 5:**
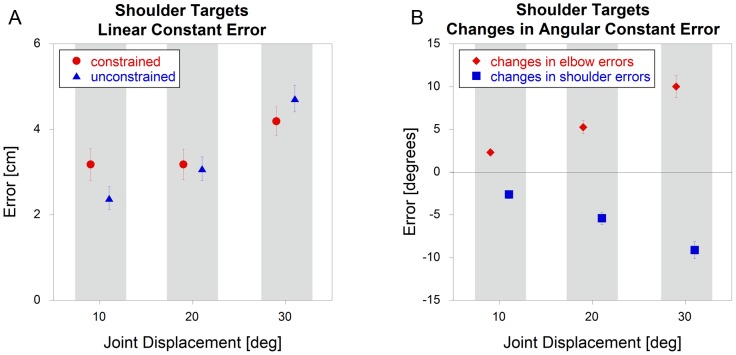
Shoulder target constant errors comparing constrained and unconstrained conditions (mean +/− sem). The data represent A) linear constant errors and B) *changes* in angular constant errors. NOTE - some of the error bars are too small to be seen.

For shoulder targets, there was also no significant effect (p = 0.57) of constraint on linear constant error (Figure 5a). However, there was a significant interaction (p = 0.020) with target angle, with follow-up t-tests indicating a significant (p = 0.015) reduction in errors under the unconstrained condition for the10 degree target. Unlike the elbow targets, when the data were analyzed in terms of joint angles, there was a significant effect (p<0.001) of constraint for both the shoulder (focal) and elbow (non-focal) joints (Figure 5b). For both joints, there was a significant interaction (p<0.001) with joint angle, with follow-up t-tests revealing a significant difference at all target angles. In all cases, unconstrained trials resulted in overshooting errors at the elbow and undershooting errors at the shoulder.

The similarity of patterns in end point accuracy between the shoulder and elbow targets can be observed by comparing figures 4a and 5a. For both, there is a gradual increase in errors at higher joint angles, but very little difference between constrained and unconstrained trials. However, there are dramatic differences when the data were analyzed in terms of joint angle, as seen by comparing figures 4b and 5b. For elbow targets, there are essentially no differences between the constrained and unconstrained conditions. However, for shoulder targets, removing the brace resulted in an increase in elbow flexion and a decrease in shoulder flexion.

### Constrained vs. Unconstrained - Variable Errors

**Figure 6:**
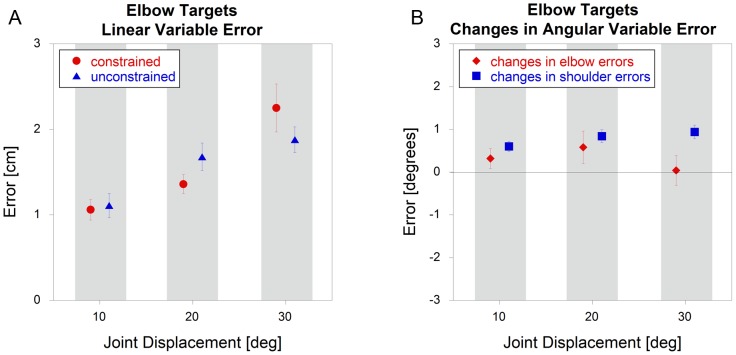
Elbow target variable errors comparing constrained and unconstrained conditions (mean +/− sem). The data represent A) linear variable errors and B) *changes* in angular variable errors.

For elbow targets, there was no significant effect (p = 0.39) of constraint on linear variable errors (Figure 6a). While there was a significant interaction with joint angle (p = 0.021), follow-up t-tests revealed no effect (p>0.20) of constraint at any target angle. When analyzed in terms of joint angles, there were no significant differences (p = 0.24) between conditions for the elbow (focal) joint, but there was a significant increase (p<0.001) in error under unconstrained conditions for the shoulder (non-focal) joint (Figure 6b).

**Figure 7:**
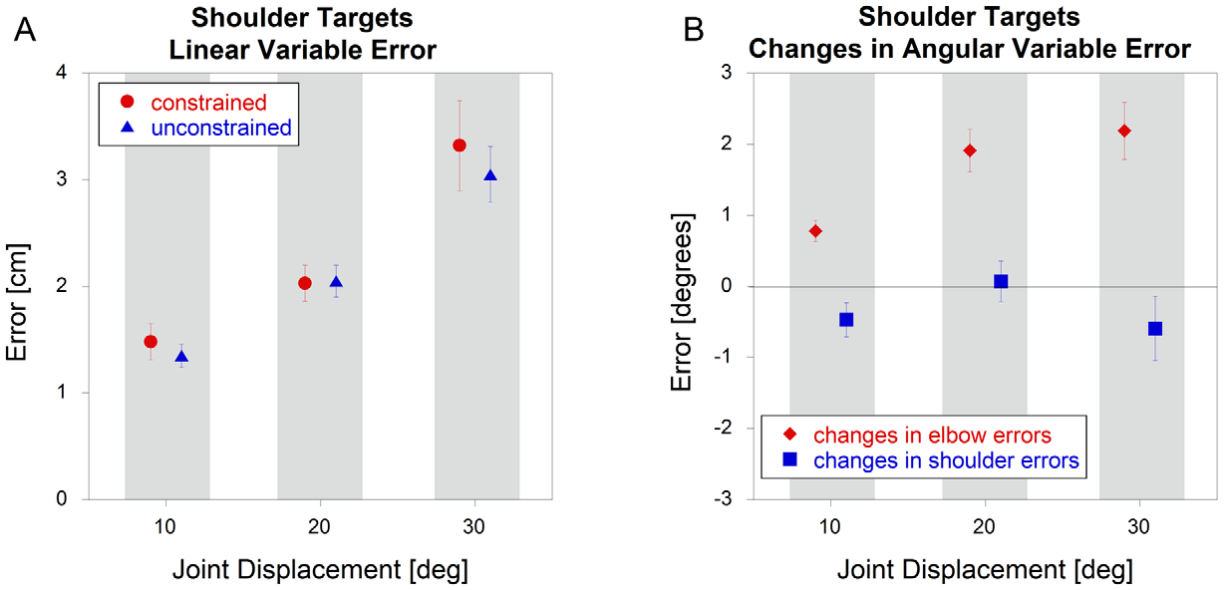
Shoulder target variable errors comparing constrained and unconstrained conditions (mean +/− sem). The data represent A) linear variable errors and B) *changes* in angular variable errors.

As with elbow targets, there was no significant effect (p = 0.97) of constraint for shoulder targets on linear variable errors (Figure 7a). The joint angle analysis was also similar to the that of the elbow: no differences (p = 0.14) between conditions for the shoulder (focal) joint, but a significant increase (p<0.001) in error under unconstrained conditions for the elbow (non-focal) joint (Figure 7b).

## Discussion

### Single Joint Motion

Due to possible differences in proprioceptive acuity, overlap in motor cortex representation and disparate anatomies, we hypothesized that there would be differences in positional accuracy between the shoulder and elbow joints. However, our results do not support this hypothesis when movements were assessed in terms of joint angular motion. This was true for both constant and variable errors, indicating that both the accuracy and precision of movements made at these two joints are similar. The variable error was found to increase with a similar pattern at both joints, as the excursion increased, with a doubling of the error going from 10 to 30 degrees of excursion. For constant error, there was a tendency to overshoot at low joint excursions and to undershoot at high joint excursions. The key observation here is that these error patterns were similar at both the shoulder and elbow. In terms of linear motion, errors at the shoulder were consistently higher than at the elbow. This can easily be explained given the similar angular results and the longer lever arm for the shoulder. These results suggest that the CNS does not compensate for the different geometrical contributions of each joint to hand motion, which results in different end point errors due to the differences in lever arms. It is particularly interesting that both variable and constant errors appeared to be driven by joint motion, and not by task relevant errors. Thus, even though the task error measured at the hand was greater for shoulder than elbow movements, the angular error was the same.

While these findings appear at odds with the vast differences in the structural and neuromuscular organization between the two joints, they provide support for the concept that movement errors may be less dependent on the peripheral anatomy of the neuromusculoskeletal system than the neural processes that represent the two segments as independent structures. This idea is consistent with research suggesting that the nervous system employs models of the body that reflect linked segment dynamics, which would depend on internal representations of the linked segments. Evidence that such models allow predictions of limb and task dynamics has been provided by studies examining aftereffects and generalization of learning environmental dynamics [26], [27], [28], [29], deafferentation studies, indicating the importance of proprioception in predicting limb dynamics [9], [30], [31], and neural recordings indicating that the CNS represents parameters such as intrinsic joint configurations and limb dynamics [32], [33], [34], [35], [36]. In fact, Gritsenko et al. [37] recently provided evidence that corticospinal commands reflect anticipation of impending dynamic interactions between the limb segments. While the structure and content of neural representations used for controlling movement remains controversial [38], [39], [40], [41], [42], [43], [44], our current findings suggest that movement-related representations of the body are segment-based, which would allow for predictions of these types of inertial responses as they propagate through the linked system.

Given our present understanding of motor control processes, we remain unable to conclusively state how the body might be represented in the brain for generating efferent command signals during control of movement. Studies examining correlations between neural activity in motor cortices and behavior have reported significant relations for many kinematic and kinetic variables [36], [41], [45], [46], [47]. However, our finding that angular errors are similar for the shoulder and elbow suggests that segments of the upper extremity might underlie this representation. Errors can arise from a variety of origins in the human motor control system, including visuomotor processes related to task performance and variations in motor commands related to noise in the processing and output system [48]. One would expect that the nature of this noise will depend on how the controlled system is represented by the CNS. For example, if the representation of the body that is used to formulate visuomotor commands is based on individual muscles, one would not expect that the joints would show similar errors, due to the vast differences in muscle architecture between the joints. However, if the joints are represented as independent units, noise that arises from the processing and command system related to planning and control of movement would be expected to be similar, under conditions in which both the kinematic freedom and requirements for control at the shoulder are limited to the same plane of movement as the elbow. This was the case for the current study, which lends credence to the idea that individual joints might reflect a representational unit for neuromusculoskeletal control.

### Multi Joint Motion

For all linear variables (constant and variable errors for both shoulder and elbow targets), there were no significant differences between the constrained and unconstrained trials. However, the results were less consistent when the data were analyzed in terms of angular variables. For the elbow targets, there was no effect of constraint on constant errors for both the elbow and shoulder. So even when subjects were free to move their shoulders, they acted as if their shoulders were still locked in place, which is consistent with previous findings by Debicki and Gribble [49]. However, this effect was very different for the shoulder targets, where there were dramatically different errors in terms of joint angles. For all conditions, the shoulder undershot its target and the elbow overshot its target. This effect became more dramatic as the excursion angles increased from 10 to 30 degrees. For example, consider the target of 30 degrees for the shoulder. While the mean target linear excursion was 28 cm for both constrained and unconstrained motions, there was only a 0.5 cm difference between conditions, which was not statistically significant. However, the unconstrained condition resulted in approximately 10 degrees less shoulder flexion and 10 degrees more elbow flexion when compared to the constrained condition.

There are several possible explanations for the differences between the shoulder and elbow errors in the unconstrained conditions. It is possible that these differences varied with the different linear excursions or the different directions of the motion, associated with the two conditions. However, the more likely explanation is that in order to rotate the upper extremity at the shoulder, the attached forearm segment must also move, resulting in elbow joint interaction torques. Galloway and Koshland [50] studied single joint motions of either the shoulder or elbow joint, in which subjects were instructed not to move the other (non-focal) joint. They found that the shoulder muscles primarily determined shoulder torques, while elbow torques were a result of elbow muscles and interaction torques. In the constrained situation in the present study, the elbow was locked at a set angle, so the brace could absorb these torques. However, in the unconstrained trials, these complex torques could have resulted in rotation of the elbow joint. In order to compensate, the CNS could either increase muscle activation around the elbow to restrict these motions or adjust shoulder motion accordingly (or both). While results from Goble et al. [51] indicate that the specific strategy chosen may be related to minimizing muscle energy expenditure, Gribble et al. [33] found that energy inefficient patterns of co-contraction may be necessary for accurate movement.

It needs to be acknowledged that the experimental model used in the present study restricts motion to the transverse plane. This means that the Cartesian coordinates of the hand have been reduced from 3 to 2 degrees of freedom. Additionally, the total angular orientations of the elbow and shoulder have been reduced from 5 to 2 degrees of freedom. While this is clearly an over simplification of how the upper extremity is used in the real world, it is an established model for answering questions related to the coordinated movement of the upper extremity [34], [50], [52], [53], [54], [55]. Additionally, it allows for the presentation of veridical targets, which would be very difficult in a completely unconstrained model. Future work could involve the use of a recently developed unsupported reaching model [56].

### Conclusions

During constrained single joint motions, subjects demonstrated similar angular accuracy and precision for the shoulder and elbow joints. This suggests that the CNS is tuned to overcome differences in proprioceptive acuity, overlap in motor cortex representation and different anatomies between these joints. During unconstrained multi-joint motions, although angle errors were higher, hand position errors remained the same as that of the constrained trials. These results support the idea that the CNS utilizes abundant degrees of freedom to compensate for the potentially different contributions to end-point errors introduced by each joint.
